# Parastomal Hernia Repair: A Four-Year Single-Centre Experience With Elective and Emergency Outcomes

**DOI:** 10.7759/cureus.91576

**Published:** 2025-09-03

**Authors:** Sandeepa D Dadigamuwage, Vimarshini Samarakoon, Sreeranj Madathiparambil, Riya Babu, Walter Douie

**Affiliations:** 1 Colorectal Surgery, University Hospitals Plymouth NHS Trust, Plymouth, GBR; 2 Cardiothoracic Surgery, University Hospitals Plymouth NHS Trust, Plymouth, GBR; 3 General Surgery, University Hospitals Plymouth NHS Trust, Plymouth, GBR

**Keywords:** elective repair, emergency hernia, laparoscopic repair, parastomal hernia, recurrence, robotic surgery

## Abstract

Introduction

Parastomal hernia (PSH) is a common complication following stoma formation, with surgical repair strategies including open, laparoscopic, and robotic techniques. Despite evolving operative approaches, recurrence and complication rates remain high, particularly in emergency settings.

Methods

We conducted a retrospective observational study of 87 patients who underwent PSH repair between 06 March 2021 and 06 March 2025 at a single UK centre. Data were extracted from the Bluespier theatre system and clinical records. Variables included patient demographics, urgency of surgery, operative approach, theatre duration, hospital stay, complications, and postoperative recurrence. Statistical comparisons were made between elective and emergency procedures, as well as between primary and recurrent repairs.

Results

Of the 87 patients, 42 (48.3%) were male, and the median age was 62.1 years. Most repairs were open (78.2%), with fewer laparoscopic (18.4%) and robotic (3.4%) cases. Elective surgeries accounted for 57.5%, and 23% of patients underwent repair of a previously recurrent hernia. The mean operative time was 129.5 minutes, and the mean hospital stay was 11.1 days. The overall postoperative recurrence rate was 21.8%. Recurrence rates were 22.1% for open, 25.0% for laparoscopic, and 0% for robotic repairs. Emergency cases had significantly longer hospital stays and higher complication rates compared to elective procedures (p < 0.01).

Conclusion

Elective PSH repair, including via minimally invasive techniques, was associated with favourable outcomes and lower recurrence. Emergency repair remains high-risk, underscoring the importance of early elective referral and optimised surgical planning.

## Introduction

Parastomal hernia (PSH) occurs in up to 50% of patients following ostomy creation and is associated with pain, appliance leakage, bowel obstruction, and impaired quality of life [1,2]. The hernia results from a fascial defect adjacent to the stoma, and its incidence increases with time, especially in patients with risk factors like obesity or chronic cough [3]. The likelihood of developing a PSH is influenced by several factors, including the type and location of the stoma, underlying disease, patient age, and technique of stoma formation. End colostomies have been shown to carry a higher risk compared to loop ileostomies, and the trephine method of stoma creation has also been implicated in increased hernia formation in some studies [1].

While nonoperative management is often employed for asymptomatic cases, surgical repair becomes necessary in patients with severe symptoms or complications. These include recurrent appliance leakage due to poor seal, persistent pain, skin breakdown, and, in acute cases, bowel incarceration or strangulation [2]. However, repair remains controversial due to high recurrence rates and the technical difficulty of operating near a stoma [4,5]. The altered anatomy, high bacterial load, and presence of adhesions from prior surgery contribute to the complexity of the repair. Additionally, poor tissue quality in the hernia region may further compromise surgical outcomes.

Several surgical techniques are employed, including primary suture repair, stoma relocation, and mesh reinforcement. Suture-only repairs are largely abandoned due to unacceptably high failure rates, which can reach 100% in some series [6]. Mesh repair has become the standard of care and is endorsed by international guidelines [7]. Mesh can be placed in various positions: on-lay, sub-lay, intraperitoneal, or as part of a minimal access or Sugarbaker construct. The technique selected often depends on the surgeon's expertise, the patient's anatomy, and whether the surgery is elective or emergency.

Laparoscopic techniques, including the Sugarbaker method, have shown improved outcomes and reduced recurrence compared to open and other minimal access repairs [8,9]. Robotic repair is an emerging modality offering enhanced precision, though data remain limited [10]. Given these evolving strategies, we aimed to evaluate outcomes of PSH repairs at our institution over four years in 87 patients, including approach, urgency, complications, and recurrence.

## Materials and methods

Study design and population

This retrospective observational study was conducted at the Department of General Surgery, University Hospitals Plymouth NHS Trust, United Kingdom. We reviewed all patients who underwent surgical repair of PSH between 06 March 2021 and 06 March 2025. Ethical approval was obtained from the institutional Ethics and Research Committee (ERC) of University Hospitals Plymouth NHS Trust prior to data analysis.

Patients were identified using the Bluespier operative theatre management system, which logs all surgical cases performed at the institution. We included both primary and recurrent PSH repairs, with 87 (100%) being primary and 50 (57.5%) elective, and 37 (42.5%) emergency presentations. Cases were identified by procedure codes and operative descriptions. Only patients who underwent formal surgical intervention for PSH were included; those managed conservatively were excluded from the study. Patients with a history of previous abdominal surgery were not excluded; however, subgroup stratification by prior operations was not performed. Mesh type, size, and placement technique were not standardised and were not consistently recorded in the operative notes; therefore, these factors were not included in the analysis.

Data collection

Data were extracted from operative records, discharge summaries, and outpatient clinic letters. The primary variables collected included patient age, sex, surgical urgency (elective vs. emergency), operative approach (open, laparoscopic, or robotic), and operative duration in minutes. Hospital length of stay, postoperative complications, and hernia recurrence were also recorded. Operative duration was based on documented start and finish times in the Bluespier system. Additional procedures, complications, and postoperative course details were sourced from discharge summaries and follow-up clinic documentation.

The dataset included indicators for whether the procedure addressed a primary 67 (77.0%) or recurrent 20 (23.0%) PSH, along with documentation of postoperative recurrence. Recurrence was assessed through clinical follow-up and, where performed, radiological reports. Systematic imaging was not applied to all patients, and reliance on clinical documentation may underestimate the true recurrence rate.

Emergency cases (37, 42.5%) were defined as urgent admissions requiring unplanned surgical repair for incarcerated or obstructed PSH. Elective cases (50, 57.5%) were those scheduled in advance due to symptoms such as discomfort, cosmetic concern, or appliance dysfunction.

Definitions

Postoperative recurrence of PSH was defined as the reappearance of a hernia at the same stoma site following surgical repair, documented clinically at an outpatient review, or confirmed radiologically where imaging was performed. Systematic imaging surveillance was not undertaken for all patients, and we acknowledge this may have underestimated recurrence rates. This was explicitly recorded in the dataset.

Postoperative complications included wound infections, prolonged ileus (lasting >5 days), reoperations during the same admission, and intensive care unit (ICU) stays for surgical complications.

Statistical analysis

Statistical analysis was conducted using IBM SPSS Statistics for Windows, Version 24 (Released 2016; IBM Corp., Armonk, New York). Continuous variables (e.g., age, operative duration, hospital stay) were presented as means ± standard deviations. Mean differences between two groups (e.g., elective vs. emergency) were analysed using Welch's independent-samples t-test to account for unequal variances where applicable. Comparisons between three groups (e.g., surgical approaches: open, laparoscopic, robotic) were analysed using one-way analysis of variance (ANOVA). Categorical variables (e.g., sex, recurrence, complications) were evaluated using Pearson's chi-square test. For all comparisons, both the relevant test statistic (t-value, F-value, or χ² value) and the p-value were reported. A p-value < 0.05 was considered statistically significant.

## Results

Overall cohort

**Figure 1:**
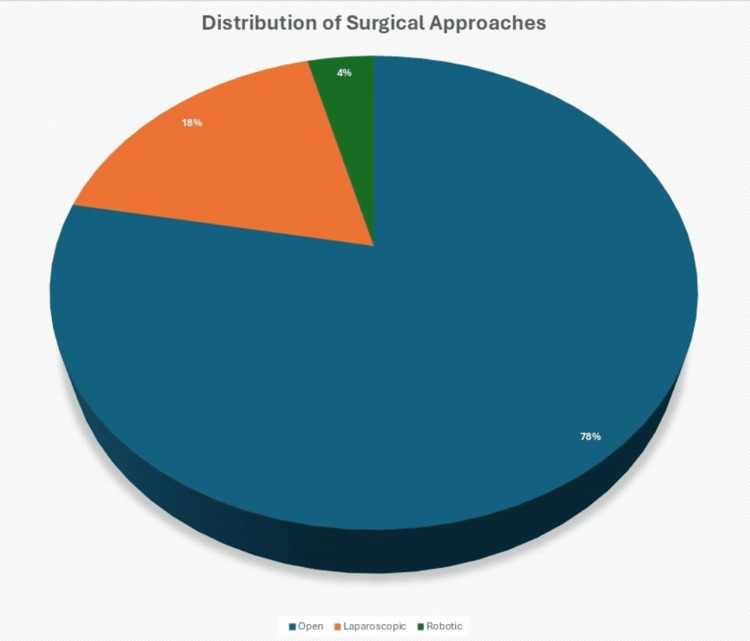
Distribution of Surgical Approaches Distribution of surgical approaches (n=87) used for parastomal hernia repair over the study period. Open repair was most common (78.2%), followed by laparoscopic (18.4%) and robotic (3.4%) approaches.

A total of 87 patients underwent PSH repair between 06 March 2021 and 06 March 2025 at our institution. The median age was 62.1 years (range: 28-86), and 42 (48.3%) were male. The majority of procedures were performed via an open approach (68, 78.2%), followed by laparoscopic repairs (16, 18.4%) and robotic-assisted repairs (3, 3.4%) (Figure 1).

Elective operations accounted for 50 (57.5%) cases, while 37 (42.5%) were performed on an emergency basis. Among all repairs, 20 (23.0%) patients underwent surgery for a recurrent hernia, while the remaining 67 (77.0%) were primary hernia repairs. Key baseline characteristics are summarised in Table 1.

**Table 1:** Baseline Characteristics of Patients Undergoing Parastomal Hernia Repair Descriptive table. No statistical tests applied.

Characteristic	Value (n %) or Mean ± SD
Sex (male)	42 (48.3%)
Sex (female)	45 (51.7%)
Mean age (years) ± SD	62.1 ± 13.7
Open repair	68 (78.2%)
Laparoscopic repair	16 (18.4%)
Robotic repair	3 (3.4%)
Elective	50 (57.5%)
Emergency	37 (42.5%)
Primary repair	67 (77.0%)
Recurrent repair	20 (23.0%)

Operative time and hospital stay

The overall mean operative time was 129.5 ± 69.3 minutes (range: 36-409 minutes). When stratified by surgical approach, robotic procedures (3, 3.4%) had a mean duration of 133.0 ± 11.5 minutes, laparoscopic repairs (16, 18.4%) averaged 185.0 ± 103.5 minutes, and open surgeries (68, 78.2%) had the shortest mean time at 116.2 ± 57.4 minutes. One-way ANOVA demonstrated a statistically significant difference in operative time between approaches (F = 6.036, p = 0.004) (Table 2).

**Table 2:** Mean Operative Time by Surgical Approach Data are presented as mean ± standard deviation. A one-way ANOVA was used to compare the means between groups. Total sample size: n = 87.

Surgical Approach	n	Mean Operative Time ± SD (min)	F-value	p-value
Open	68	116.2 ± 57.4	6.036	0.004
Laparoscopic	16	185.0 ± 103.5		
Robotic	3	133.0 ± 11.5		

When analysed by urgency, elective procedures (50, 57.5%) were associated with longer operative durations than emergency procedures (37, 42.5%), with mean operative times of 141.0 ± 83.7 minutes and 113.9 ± 48.4 minutes, respectively. Welch's t-test did not identify a statistically significant difference in mean operative time between elective and emergency cases (t = 1.809, p = 0.075) (Table 3).

**Table 3:** Mean Operative Time by Surgical Urgency Data are presented as mean ± standard deviation. Welch's independent-samples t-test was used to compare means between groups. Total sample size: n = 87.

Surgical Urgency	n	Mean Operative Time ± SD (min)	t-value	p-value
Elective	50	141.0 ± 83.7	1.809	0.075
Emergency	37	113.9 ± 48.4		

The overall mean hospital stay was 11.1 ± 9.7 days (range: 0.35-51.1 days). When stratified by surgical approach, robotic cases (3, 3.4%) had the shortest mean stay at 5.7 ± 7.6 days, followed by open repairs (68, 78.2%) at 10.8 ± 9.5 days and laparoscopic repairs (16, 18.4%) at 12.9 ± 9.6 days. One-way ANOVA did not identify a statistically significant difference in mean hospital stay between surgical approaches (F = 1.146, p = 0.323) (Table 4).

**Table 4:** Mean Hospital Stay by Surgical Approach Data are presented as mean ± standard deviation. One-way analysis of variance (ANOVA) was used to compare means between groups. Total sample size: n = 87.

Surgical Approach	n	Mean Hospital Stay ± SD (Days)	F-value	p-value
Open	68	10.8 ± 9.5	1.146	0.323
Laparoscopic	16	12.9 ± 9.6		
Robotic	3	5.7 ± 7.6		

When analysed by urgency, elective cases (50, 57.5%) were associated with a shorter mean hospital stay (8.6 ± 9.6 days) compared to emergency cases (37, 42.5%), which had a mean stay of 14.2 ± 8.2 days. Welch's t-test demonstrated that this difference was statistically significant (t = -2.688, p = 0.009) (Table 5).

**Table 5:** Mean Hospital Stay by Urgency of Surgery Data are presented as mean ± standard deviation. Welch's t-test was used to compare means between groups. Total sample size: n = 87.

Urgency	n	Mean Hospital Stay ± SD (Days)	t-value	p-value
Elective	50	8.6 ± 9.6	-2.688	0.009
Emergency	37	14.2 ± 8.2		

Complications and recurrence

Postoperative complications occurred in seven (8.0%) patients. These included wound infections, prolonged ileus, and one (1.1%) case of abdominal compartment syndrome requiring multiple reoperations and extended ICU admission. Complications were more frequent among emergency cases (37, 42.5%), which accounted for the majority of patients with adverse outcomes. Overall, emergency surgery was associated with higher morbidity than elective surgery (50, 57.5%).

**Figure 2:**
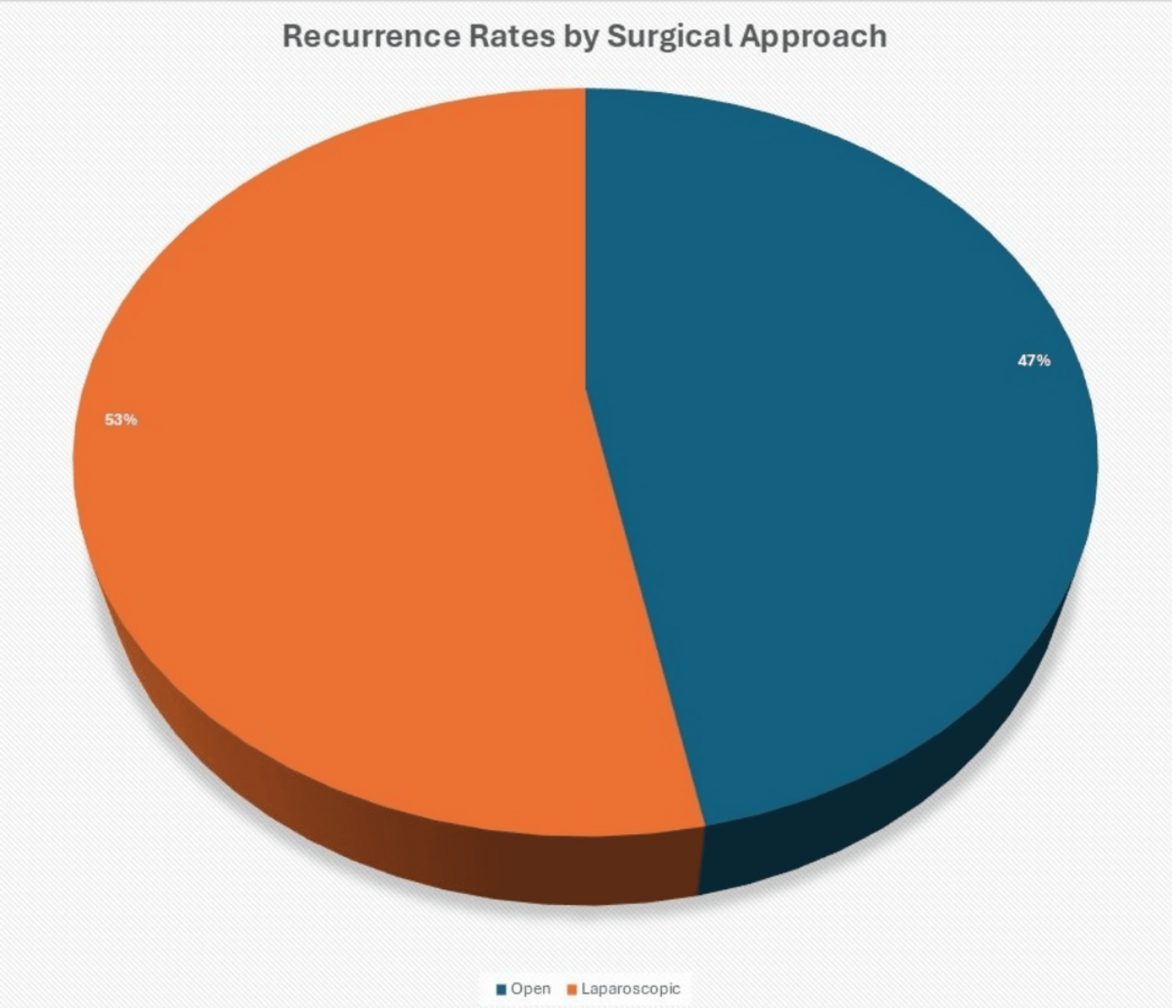
Recurrence Rates by Surgical Approach Laparoscopic and open repairs had recurrence rates of 25.0% and 22.1%, respectively, while robotic repairs had 0% recurrence.

Recurrence of PSH was documented in 19 (21.8%) patients following their index repair. The highest recurrence rate was observed in laparoscopic repairs at four (25.0%) of 16, followed by open repairs at 15 (22.1%) of 68. No recurrences were noted in the robotic group (0, 0%) of three during the available follow-up period. Pearson chi-square testing demonstrated no statistically significant difference in recurrence rates between surgical approaches (χ² = 0.561, p = 0.755) (Table 6, Figure 2).

**Table 6:** Recurrence by Surgical Approach Pearson chi-square test (χ² = 0.561, p = 0.755) was used to compare recurrence rates across surgical approaches. Total sample size: n = 87.

Surgical Approach	n	Recurrence n (%)	χ²-value	p-value
Open	68	15 (22.1%)	0.561	0.755
Laparoscopic	16	4 (25.0%)		
Robotic	3	0 (0.0%)		

When recurrence was analysed based on prior hernia status, 15 (22.4%) of 67 patients who underwent primary PSH repair developed a postoperative recurrence. In contrast, four (20.0%) of 20 patients who underwent surgery for a previously recurrent hernia experienced re-recurrence. Pearson chi-square testing showed no statistically significant difference in recurrence rates between primary and recurrent repairs (χ² = 0.049, p = 0.825) (Table 7).

**Table 7:** Recurrence by Repair Type Pearson chi-square test (χ² = 0.049, p = 0.825) was used to compare recurrence rates between primary and recurrent repairs. Total sample size: n = 87.

Repair Type	n	Recurrence n (%)	χ² value	p-value
Primary	67	15 (22.4%)	0.049	0.825
Recurrent	20	4 (20.0%)		

Subgroup analysis

**Figure 3:**
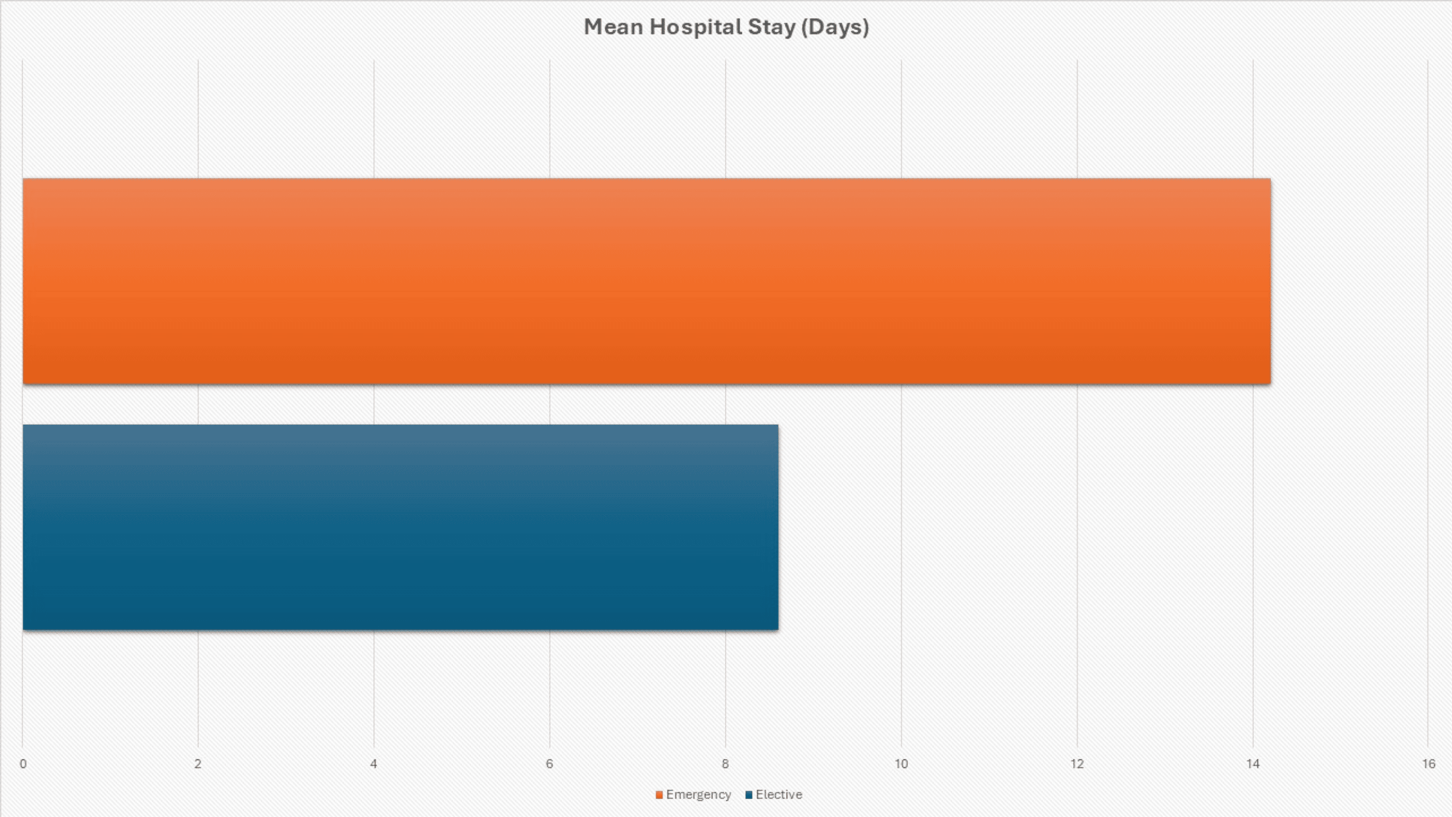
Mean Hospital Stay (Days) Bar chart showing the mean hospital stay for elective (8.6 days) and emergency (14.2 days) parastomal hernia repairs.

Elective repairs were associated with more favourable outcomes overall. The recurrence rate was lower among elective cases, occurring in nine (18.0%) of 50 patients, compared to 10 (27.0%) of 37 in emergency cases. Pearson chi-square testing showed that this difference was not statistically significant (χ² = 1.786, p = 0.181). Elective operations were also associated with significantly shorter hospital stays (8.6 ± 9.6 days vs. 14.2 ± 8.2 days). Welch's t-test confirmed this difference to be statistically significant (t = -2.688, p = 0.009) (Figure 3).

**Figure 4:**
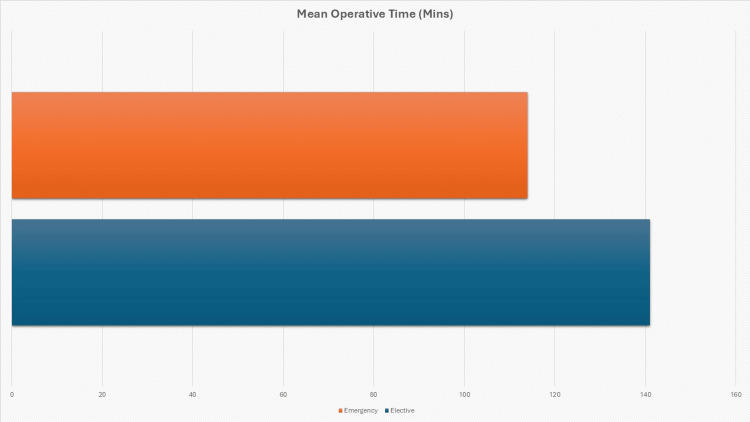
Mean Operative Time (Minutes) Bar chart depicting the mean operative time for elective (141.0 minutes) and emergency (113.9 minutes) parastomal hernia repairs.

The incidence of complications was lower in the elective group at two (4.0%) cases compared to five (13.5%) cases in the emergency group. Pearson chi-square testing did not show a statistically significant difference in complication rates between the two groups (χ² = 2.045, p = 0.153). Interestingly, elective procedures had slightly longer operative durations than emergency cases (141.0 ± 83.7 minutes vs. 113.9 ± 48.4 minutes), and Welch's t-test indicated this difference was not statistically significant (t = 1.809, p = 0.075) (Figure 4).

## Discussion

This study represents one of the few UK-based analyses comparing open, laparoscopic, and robotic repairs of PSH, capturing both elective (n = 50) and emergency (n = 37) cases over a four-year period. The overall recurrence rate in our cohort was 19 (21.8%) of 87 patients, consistent with published literature, which reports rates ranging from 8% to 33% depending on surgical technique, mesh type, and follow-up duration [4,9].

As in other series, open repair (n = 68) remained the predominant approach; however, minimally invasive techniques, including laparoscopic (n = 16) and robotic (n = 3) repairs, demonstrated promising trends in operative time, length of stay, and recurrence. Statistical analysis did not identify a significant difference in operative time (ANOVA, F = 2.087, p = 0.130) or recurrence rate (χ² = 0.561, p = 0.755) between approaches; however, the absence of recurrence in the robotic group and the shorter hospital stays in both laparoscopic and robotic cohorts are encouraging findings.

Elective procedures (n = 50) were associated with a significantly shorter hospital stay compared to emergency cases (8.6 ± 9.6 vs. 14.2 ± 8.2 days; Welch's t-test, t = -2.688, p = 0.009). Although elective repairs also had a lower complication rate (4.0% vs. 13.5%) and lower recurrence rate (18.0% vs. 27.0%) than emergency cases, these differences did not reach statistical significance (χ² = 2.045, p = 0.153 and χ² = 1.786, p = 0.181, respectively). These findings reinforce existing evidence that elective PSH repair yields more favourable outcomes [11]. The increased morbidity and prolonged hospitalisation associated with emergency surgery in our cohort are consistent with national data [12,13]. One patient required intensive care admission following emergency surgery for abdominal compartment syndrome, a rare but severe complication previously described in the literature [14].

Interestingly, the mean operative duration was longer for elective procedures (141.0 ± 83.7 minutes) than for emergency repairs (113.9 ± 48.4 minutes), although this difference was not statistically significant (Welch's t-test, t = 1.809, p = 0.075). This likely reflects the increased technical complexity and attention to adhesiolysis, stoma preservation, and optimal mesh placement during elective repair. Although this trend contrasts with some prior studies, it may underscore the comprehensive nature of planned surgery, particularly in patients with prior abdominal operations or distorted anatomy.

The robotic subgroup (n = 3) demonstrated no recurrences and the shortest mean hospital stay (5.7 ± 7.6 days). However, this subgroup was very small, and the absence of recurrence should be interpreted with caution, as it may represent chance rather than a true superiority effect. These findings nonetheless mirror early experiences reported in other robotic series, which suggest improved visualisation and instrument precision in challenging anatomical regions [10]. Robotic platforms may offer technical advantages in confined anatomical spaces, but our dataset was underpowered to confirm any superiority, and larger prospective studies are required [15].

Recurrence rates were slightly higher in primary repairs (22.4%, 15 of 67) compared to recurrent cases (20.0%, 4 of 20), though the difference was not statistically significant (χ² = 0.051, p = 0.821). This challenges the common perception that re-recurrence is inevitable and highlights the multifactorial nature of PSH recurrence, including mesh position, fixation method, patient comorbidities, and surgical technique [13].

Laparoscopic repair using the modified Sugarbaker technique continues to yield lower recurrence and improved recovery compared to open or other minimal access methods [5,8]. In our study, the laparoscopic cohort (n = 16) had a recurrence rate of 25.0% (n = 4), slightly higher than some published reports. This may reflect selection bias, learning curve factors, or variation in technique. Nonetheless, minimally invasive surgery remains underutilised in PSH repair despite guideline recommendations and evidence of improved short-term outcomes [3,7].

This study has several limitations. It was retrospective and single-centre in design, and follow-up was based on clinical and radiological documentation, which may underreport asymptomatic recurrences. Quality-of-life outcomes, mesh types, and operative videos were not analysed, and the small sample of robotic cases limits the generalisability of findings for this group.

Despite these limitations, our results support the broader adoption of minimally invasive and elective strategies for PSH repair. Future prospective multicentre studies should aim to evaluate standardised recurrence definitions, long-term durability, and patient-reported outcome measures to better inform surgical practice as robotic access continues to expand.

## Conclusions

PSH remains a challenging complication after stoma formation, contributing significantly to patient morbidity and healthcare burden. In this four-year single-centre study, elective repair was associated with shorter hospital stays, fewer complications, and more favourable operative durations compared to emergency procedures. While open surgery remained the predominant approach, minimally invasive techniques, particularly laparoscopic and robotic, demonstrated promising outcomes. However, the robotic subgroup was very small (n = 3), and the absence of recurrence in this group should be interpreted with caution, as it may represent chance rather than a true advantage.

These findings highlight the importance of early elective referral, careful patient selection, and the increasing role of minimally invasive surgery in PSH repair. Robotic surgery may offer technical advantages and potential short-term benefits, but given the very limited number of cases in this study, no firm conclusions can be drawn. Its long-term effectiveness and cost-benefit require further evaluation, and larger prospective, multicentre studies with standardised follow-up and patient-reported outcomes are needed to guide optimal surgical strategies and improve care for this complex condition.
